# Unveiling the complementariness of robotic tablet dispensing machines for elderly care: A bibliometric data analysis

**DOI:** 10.1016/j.rcsop.2024.100545

**Published:** 2024-11-26

**Authors:** Sunday Adewale Olaleye, Olaleye Esther Olubunmi, Berhanemeskel Weldegerima Atsbeha, Mulugeta Negash Wodaje

**Affiliations:** a College of Business and Economics, University of Gondar, P.O. Box 196, Gondar, Ethiopia; b Smartstep Consultancy, 16, Bello Street, Off Precious Seed, Onibuku, Ota, Ogun State, Nigeria; c Department of Social and Administrative Pharmacy, School of Pharmacy, University of Gondar, P.O. Box 196, Gondar, Ethiopia; d Department of Marketing Management, University of Gondar, P.O. Box 196, Gondar, Ethiopia.

**Keywords:** Robotic assistance, Automatic pill dispenser, Medication management, Adherence, Healthcare

## Abstract

This study conducts a bibliometric analysis of academic papers and conference proceedings related to tablet dispensers, medicine dispensers, and pill dispensers within the framework of Sustainable Development Goal 03: Good Health and Well-Being. The analysis spans literature published between 1997 and 2023. Utilizing the Web of Science database, the study employs keywords such as “tablet dispenser,” “medicine dispenser,” and “pill dispenser” to gather relevant English-language papers classified as Proceeding Papers or Articles. The inclusion and exclusion criteria filtered 79 initial records down to 40, focusing on articles pertinent to SDG 03. Data analysis was performed using the Biblioshiny App through RStudio, examining publication trends, authorship patterns, citation networks, and other bibliometric indicators. The findings reveal a steady increase in research output, moderate citation impact, extensive references, and collaborative authorship, with limited international collaboration. The study underscores the growing interest and research activity in robotic tablet dispensing machines for elderly care while highlighting areas for further global engagement.

## 1 Introduction

Global automatic pill dispenser machines or robot drug dispensers are disrupted, but Africa needs to catch up. The automated drug machine market has witnessed significant growth in recent years, with a valuation of $2.5 billion in 2020. Projections suggest this figure will nearly double, reaching $5.4 billion by 2030, exhibiting a Compound Annual Growth Rate (CAGR) of 8.2 % spanning 2021 to 2030.^1^ Automatic pill dispensers, devices designed to distribute medication at predetermined times, have emerged as essential tools in healthcare settings, particularly for individuals facing challenges in adhering to their prescribed medication schedules, such as seniors and those with chronic illnesses.

These devices, commonly used in medical facilities, aim to ensure timely and accurate administration of prescribed medications, over-the-counter drugs, and supplements.^2^ Moreover, advancements in telehealth technology have facilitated the integration of automatic pill dispensers into remote patient monitoring systems, enabling healthcare providers to monitor and manage medication adherence remotely.^3^

The growth trajectory of the automatic pill dispenser machine market is fueled by several factors, including the increasing demand for medication intake, particularly among the aging population, and the prevalence of diseases associated with old age.^4^ Furthermore, the rise in chronic and infectious diseases worldwide and continuous technological innovations to enhance the efficacy of automatic pill dispenser machines further drive market growth.^5^

Despite these favourable market dynamics, stringent government regulations governing the manufacturing and sale of automatic pill dispenser machines pose challenges to market expansion.^6^ However, the increasing disposable income of consumers presents lucrative opportunities for key players in the pill dispenser market to capitalize on the breakthrough.

The automatic pill dispenser machine is based on product type, application, and geographic region market segments^7^ Product categories include centralized, automated dispensing systems and decentralized automated dispensing systems, while applications span hospital pharmacies, retail pharmacies, and home healthcare settings.^8^ The market is analyzed geographically across North America, Europe, Asia-Pacific, and LAMEA (Latin America, Middle East, and Africa), reflecting the global scope and regional variations in market dynamics.^9^

The wrong dosage of tablets among seniors is a continuous challenge in elderly homes or hospitals. These drug dispensing errors can occur due to the wrong perception of similar drug names or incorrect quantity dosages^10^, ^11^, 12., 13. One potential solution to this long-standing problem in the medical sector is a robotic tablet dispensing machine, an offshoot of personal digital assistance (PDA) technology.^12^ This technology disruption in the medical sector is spreading globally. A liquid medication dispensing robot has also been introduced as a panacea for patients with swallowing difficulties.^14^ While the adoption of robotic tablet dispensing machines is still in progress in many countries worldwide,^15^ other countries are already at the forefront of using this technology, with recent studies projecting its future developments.^16^

Due to the gaps in the literature of tablet dispenser, this study analyses relevant literature on robotic tablet dispensing machines to assess progress and setbacks using various bibliometric indicators. Despite the gradual growth of literature in robotic research, specifically robotic tablet dispensers, it is paramount to identify trends, map the research landscape, benchmark performance, and gain a deep understanding of this research focus. This understanding can inform innovation and development and support decision-making for managers of robotic tablet dispensing machines. The study divides into theoretical backgrounds, methodology, discussion and conclusions with theoretical, managerial implications and future research insights.

The Complementariness of Robotic Tablet Dispensing Machines for Elderly Care and Sustainable Development Goals - Good Health and Wellbeing. The global population is rapidly aging, and with it comes an increased demand for effective elderly care solutions.^17^ Simultaneously, the world strives to achieve the Sustainable Development Goals (SDGs), with one of the critical targets being good health and well-being.^18^ In this context, integrating robotic tablet dispensing machines for elderly care has emerged as a promising solution that aligns with the SDGs. This historic overview aims to provide an academic analysis of the evolution and implications of robotic tablet dispensing machines in the context of elderly care and their contribution to achieving the SDGs, particularly the goal of good health and well-being.

The use of technology in healthcare has a long history, with significant advancements in recent decades.^19^ Robotic technologies have gained prominence due to their potential to enhance efficiency, accuracy, and patient care.^20^ The development of robotic tablet dispensing machines specifically designed for elderly care can be traced back to the early 1970s.^21^ These machines were initially introduced to improve medication management and adherence for older adults living independently or in assisted living facilities.^22^

Over time, robotic tablet dispensing machines have evolved from simple medication reminder devices to sophisticated systems capable of automated medication dispensing, monitoring, and communication with healthcare providers.^23^ Integrating artificial intelligence (AI) and machine learning algorithms has further enhanced their capabilities, enabling personalized medication schedules, real-time medication intake monitoring, and remote access for healthcare professionals.^16^

The introduction of robotic tablet dispensing machines has significantly improved elderly care by ensuring reliable medication adherence, thereby reducing the risk of missed doses or incorrect administration. This advancement is particularly crucial for older adults who often have complex medication regimens. Additionally, automating medication dispensing alleviates the burden on caregivers and healthcare professionals, enabling them to concentrate on other aspects of care. Moreover, the real-time monitoring and communication features of these machines allow for early detection of medication non-compliance or adverse reactions, facilitating timely interventions and preventing potential health complications.

Integrating robotic tablet dispensing machines for elderly care aligns closely with the SDGs, particularly the goal of good health and well-being. By improving medication management and adherence, these machines contribute to the prevention and control of chronic diseases, significantly contributing to the global disease burden. Additionally, the automation and remote monitoring capabilities reduce unnecessary hospital visits, promoting efficient healthcare resource utilization and reducing healthcare costs. Furthermore, using AI and machine learning in these machines allows for continuous learning and improvement, leading to personalized and optimized care for the elderly.

The historical overview of the mix of robotic tablet dispensing machines for elderly care and the SDGs highlights the evolution and benefits of these technologies. Integrating robotic tablet dispensing machines has revolutionized medication management for older adults, ensuring adherence, reducing caregiver burden, and enabling real-time monitoring. Furthermore, these machines align with the SDGs, particularly the goal of good health and well-being, by promoting efficient healthcare resource utilization and personalized care. As the global population ages, adopting robotic tablet dispensing machines holds great promise for improving elderly care and achieving the SDGs.

## 2 Theoretical background

### 2.1 Human-robot interaction (HRI) theory

Human-robot interaction (HRI) has emerged as a significant research area in recent years, focusing on understanding and improving interactions between humans and robots.^24^ This field has gained substantial attention due to the increasing integration of robots into various domains of human life, including healthcare, education, and entertainment. Developing effective HRI systems is crucial for ensuring seamless communication, intuitive interfaces, and user-friendly experiences. This literature review explores the existing research on HRI theory, particularly in the context of medication information communication, reminders, and user queries.

One of the critical goals of HRI theory is to design robotic systems that can effectively communicate medication information to users. Medication adherence is a critical aspect of healthcare, and using robots in this domain has shown promising results. For instance, a study by Datta et al.^25^ investigated using a social robot to deliver medication reminders to older adults. The results indicated that the robot's reminders significantly improved medication adherence compared to traditional methods. This progress highlights the potential of HRI theory in designing robots that can effectively communicate medication information and enhance patient outcomes.

In addition to medication reminders, HRI theory also focuses on developing robots that can respond to user queries in an intuitive and user-friendly manner. Natural language processing and dialogue systems are crucial in achieving this goal. Li et al.^26^ explored using a dialogue system in a social robot to answer user queries related to medication. The results demonstrated that the robot's ability to provide accurate and relevant information significantly improved user satisfaction and engagement. This research emphasizes the importance of HRI theory in designing robots that can effectively respond to user queries and provide relevant information.

Furthermore, HRI theory also emphasizes the importance of intuitive and user-friendly interfaces in robotic systems. The design of interfaces plays a crucial role in facilitating effective communication and interaction between humans and robots. A study by Chang et al.^27^ investigated the impact of different interface designs on user engagement and trust in a social robot. The results indicated a visually appealing and user-friendly interface significantly enhanced user engagement and trust. This development highlights the significance of HRI theory in guiding the design of robotic interfaces to ensure a positive user experience.

It is important to note that while HRI theory has shown promising results in various domains, challenges still need to be addressed. One of the key challenges is the need for personalized interactions. Every individual has unique preferences, communication styles, and needs. Designing robotic systems that can adapt to these individual differences is a complex task. Robinson et al.^28^ explored personalized interaction strategies in a social robot for medication reminders. The results indicated that personalized interactions significantly improved user engagement and satisfaction. This research highlights the importance of considering individual differences in HRI theory to enhance the effectiveness of robotic systems.

Another challenge in HRI theory is the ethical considerations associated with robot-human interactions. As robots become more integrated into human life, ethical issues such as privacy, autonomy, and trust must be carefully addressed. A study by Fink et al.^29^ discussed the ethical implications of using social robots in healthcare settings. The research emphasized the importance of ethical guidelines and regulations to ensure the responsible and safe use of robots in human interactions. This safety issue shows the need for HRI theory to not only focus on technical aspects but also consider the ethical implications of robotic systems.

HRI theory is crucial in understanding and improving interactions between humans and robots. The development of robotic systems that effectively communicate medication information, provide reminders, respond to user queries intuitively, and are user-friendly is essential for enhancing patient outcomes and user satisfaction.

### 2.2 Health belief model (HBM)

The Health Belief Model (HBM) is a widely recognized theoretical framework that aims to understand and predict individuals' health-related behaviours.^30^, ^31^, ^32^ According to the HBM, these behaviours are influenced by four key factors: perceptions of susceptibility to illness, the severity of the illness, benefits of acting, and barriers to taking action. In recent years, robotic assistance in medication management has emerged as a potential solution to address these factors and promote medication adherence. This literature review will explore the existing research on the use of robotic assistance in medication management and its potential impact on individuals' health beliefs and behaviours.

Perceptions of susceptibility to illness play a crucial role in motivating individuals to engage in health-related behaviours. Research has shown that individuals who perceive themselves as at a higher risk of developing an illness are more likely to take preventive actions.^33^ Robotic medication management assistance can enhance individuals' perceptions of susceptibility by providing timely reminders and personalized medication schedules. For instance, a robotic device can remind individuals to take their medications at specific times, increasing their awareness of the importance of medication adherence and their susceptibility to potential health risks if they fail to do so.

The severity of the illness is another crucial factor influencing individuals' health behaviours. When individuals perceive an illness to be severe, they are more likely to take action to prevent or manage it.^34^ Robotic assistance in medication management can help individuals understand the severity of their illness by providing educational information about the consequences of non-adherence. For example, a robotic device can display visual representations or provide audio explanations of the potential complications of not taking medications as prescribed. This situation can enhance individuals' perception of the severity of their condition and motivate them to adhere to their medication regimen.

The benefits of taking action are crucial in influencing individuals' health behaviours. If individuals perceive that the benefits of engaging in a particular behavior outweigh the costs or efforts required, they are more likely to adopt and maintain that behavior.^34^ Robotic assistance in medication management can highlight the benefits of medication adherence by providing educational information about the positive outcomes associated with proper medication use. For instance, a robotic device can present statistics or success stories of individuals who have experienced improved health outcomes due to medication adherence. By emphasizing the potential benefits, robotic assistance can enhance individuals' motivation to adhere to their medication regimen.

Barriers to taking action, such as forgetfulness or the complexity of medication regimens, can hinder individuals' engagement in health behaviours. Robotic assistance in medication management can help overcome these barriers by providing timely reminders and personalized medication schedules. For example, a robotic device can send notifications or alarms to remind individuals to take their medications at the prescribed times. Additionally, it can simplify medication regimens by organizing and dispensing medications in a user-friendly manner. By addressing these barriers, robotic assistance can facilitate individuals' adherence to their medication regimen.

In conclusion, the Health Belief Model provides a valuable framework for understanding individuals' health behaviours. Robotic assistance in medication management has the potential to address the critical factors identified in the HBM by providing timely reminders, personalized medication schedules, and educational information about the importance of medication adherence. Robotic assistance can promote medication adherence and ultimately improve health outcomes by enhancing individuals' perceptions of susceptibility, severity, benefits, and barriers.

## 3 Methodology

This study's purpose was to conduct a bibliometric analysis of academic papers and conference proceedings related to tablet dispensers, medicine dispensers, and pill dispensers within the context of Sustainable Development Goal 03: Good Health and Well-Being. The study aimed to identify and analyze the relevant literature published between 1997 and 2023. This section describes the methodology employed to conduct the bibliometric analysis, including the search strategy, inclusion and exclusion criteria, and data analysis.

### 3.1 Search strategy

The study utilized the Web of Science database on January 22, 2024, to identify relevant academic papers and conference proceedings. The search was conducted using the keywords “tablet dispenser,” “medicine dispenser,” and “pill dispenser.” These keywords were selected to ensure a comprehensive search, encompassing various terminologies used in the literature. The search was limited to papers published in English and classified as either Proceeding Paper or Article. The publication years included were from 1997 to 2023.

### 3.2 Inclusion and exclusion criteria

The inclusion and exclusion criteria were established to ensure that only relevant literature related to Sustainable Development Goal 03: Good Health and Well-Being was included in the analysis. The initial search generated 79 academic papers. One Spanish-language publication was excluded, resulting in 78 academic records written in English. The search was further refined by excluding meeting abstracts (2), news items (2), and academic papers (34) that were outside the scope of SDG 03. This process left a final selection of 16 articles and 24 conference proceedings, totalling 40 papers for data analysis.

### 3.3 Data analysis

The selected academic papers and conference proceedings were subjected to quantitative data analysis using the Biblioshiny App through the RStudio platform. The Biblioshiny App is a powerful tool for bibliometric analysis that allows for extracting relevant data from scientific literature. It provides various metrics and visualizations to analyze publication trends, authorship patterns, citation networks, and other bibliometric indicators.

The data analysis involved extracting relevant information from the selected papers, such as publication year, author names, affiliations, journal or conference information, keywords, and citation counts. The extracted data were then analyzed using descriptive statistics and visualizations to identify patterns, trends, and relationships within the literature. This analysis provided insights into the research landscape, the key contributors, and the research gaps in tablet, medicine, and pill dispensers within the context of SDG 03.

### 3.4 Prisma flow chart

A Prisma flow chart was constructed to visually represent the inclusion and exclusion process of the literature search. The flow chart illustrates the number of papers identified, screened, and included in the final selection. It provides transparency and clarity regarding the study's methodology and ensures that the selection process is replicable by other researchers.

**Fig. 1:**
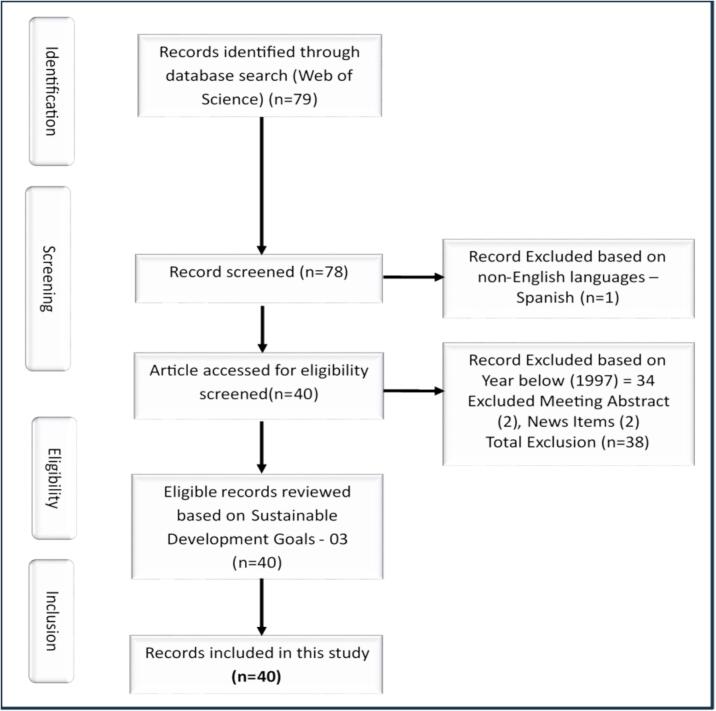
Prisma flow chart of inclusion and exclusion.

Fig. 1 presents the Prisma flow chart for this study. It shows the number of papers at each stage of the inclusion and exclusion process, including the initial search, the exclusion of non-English publications, the exclusion of papers outside the scope of SDG 03, and the final selection of papers for data analysis.

The methodology employed in this study involved a systematic search strategy, rigorous inclusion and exclusion criteria, and quantitative data analysis using the Biblioshiny App. The search generated 79 academic papers, refined to a final selection of 40 papers for data analysis. The Prisma flow chart provides a clear overview of the inclusion and exclusion process, ensuring transparency and replicability of the study. The following section will present the findings and discussion based on the data analysis of the selected papers.

The study utilized Web of Science on 22.01.2024 and initially searched for “tablet dispenser” (Topic) or “medicine dispenser” (Topic) or “pill dispenser” (Topic) and limited the publication years to 2023, 2022, 2021, 2020, 2019, 2018, 2017, 2016, 2015, 2014, 2013, 2012, 2010, 2009, 2008, 2007, 2006, 1999, 1998, or 1997 (Publication Years) in English (Languages) and classified as Proceeding Paper or Article (Document Types) related to Sustainable Development Goals (SDG 03) as an extension of Good Health and Well Being. The search generated 79 academic papers. After excluding one Spanish-language publication, 78 academic records written in English remained. The search was limited to 1997 to 2023 and focused on Sustainable Development Goal 03: Good Health and Wellbeing. Meeting abstracts (2) and news items (2), and (34) academic papers outside the scope of SDG were excluded, leaving a final selection of 16 articles and 24 conference proceedings, totaling 40 papers for data analysis using the quantitative Biblioshiny App through the RStudio platform.

## 4 Results

### 4.1 Descriptive Statistics of the Robotic Dispense Machine

**Fig. 2:**
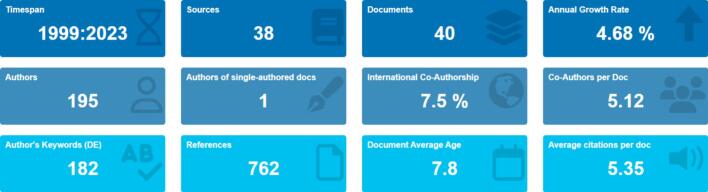
Descriptive statistics of the Robotic Dispense Machine.

In this section based on Fig. 2 presents the descriptive statistics of the Robotic Dispense Machine. The descriptives cover a time span from 1999 to 2023 and include 40 documents sourced from 38 journals. The annual growth rate of the documents in this field is 4.68 %, indicating a steady increase in research output over time. The average age of the documents in this analysis is 7.8 years, suggesting that the research in this area is relatively recent. On average, each document in this dataset receives 5.35 citations, indicating a moderate level of impact and recognition within the scholarly community.

The documents' references section in this analysis contains 762 citations, indicating that the authors have extensively reviewed the existing literature on the topic. This description suggests a comprehensive understanding of the field and a strong foundation for the research presented in these documents. The document contents were analyzed in terms of keywords. The analysis revealed the presence of 112 Keywords Plus (ID) and 182 Author's Keywords (DE). These keywords provide insights into the main themes and concepts explored in the literature on robotic tablet dispensing machines for elderly care.

The analysis also examined the characteristics of the authors contributing to this field. A total of 195 authors were identified, with only one single-authored document. It indicates a high level of collaboration among researchers in this area. On average, each document has 5.12 co-authors, suggesting a multidisciplinary approach and a diverse range of expertise contributing to the research. Furthermore, the analysis explored international collaboration among authors. Only 7.5 % of the documents included international co-authorships. This result indicates that most of the research in this field is conducted within national boundaries, with limited international collaboration.

This analysis categorized the document types as articles and proceedings papers. Of the 40 documents, 16 were articles, and 24 were proceedings papers. This result reveals that researchers prefer to disseminate their findings through conference proceedings, indicating an active engagement in academic conferences and professional gatherings.

The results of the bibliometric analysis indicate a growing interest in the topic of robotic tablet dispensing machines for elderly care. The research output in this area has steadily increased over the years, with moderate impact and recognition within the scholarly community. The extensive references and keywords used in the documents demonstrate a comprehensive understanding of the field. The high level of collaboration among authors and the preference for conference proceedings as a mode of dissemination suggests an active and engaged research community. However, the limited international collaboration highlights the need for further global engagement and exchange of ideas in this field.

### 4.2 Author's production over time

**Fig. 3:**
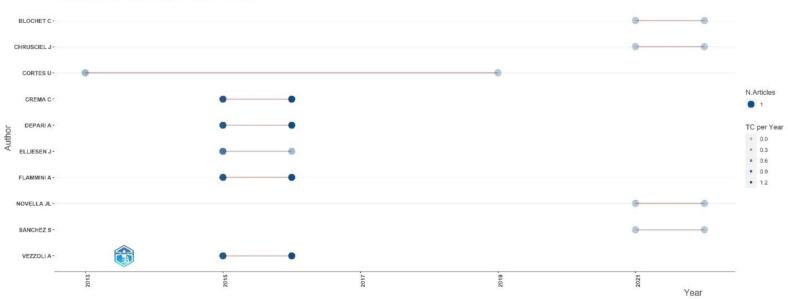
Author's production over time.

Fig. 3 shows the author's production over time and revealed a varied range of research topics, with some articles receiving citations while others have yet to receive any. The lack of citations for most articles suggests they may have had a minor impact on the field. However, it is essential to note that citation counts alone do not necessarily reflect the quality or impact of a publication. Further analysis and evaluation of the content and contributions of each article would be necessary to assess the author's production fully.

The bibliometric data analysis in Table 1, reveals varied academic contributions and their impact over different years. Several authors, including BLOCHET C, CHRUSCIEL J, NOVELLA JL, and SANCHEZ S, have published recent works in 2021 and 2022, but these have not yet received any citations, indicating a lack of immediate impact. In contrast, authors like CREMA C, DEPARI A, FLAMMINI A, and VEZZOLI A have shown sustained academic influence with publications from 2015 and 2016, each receiving 11 citations, and demonstrating consistent citation rates over time.

**Table 1:** Author's production over time.

Author	Year	Freq	TC	TCpY
BLOCHET C	2021	1	0	0
BLOCHET C	2022	1	0	0
CHRUSCIEL J	2021	1	0	0
CHRUSCIEL J	2022	1	0	0
CORTES U	2013	1	2	0.167
CORTES U	2019	1	0	0
CREMA C	2015	1	11	1.1
CREMA C	2016	1	11	1.222
DEPARI A	2015	1	11	1.1
DEPARI A	2016	1	11	1.222
ELLIESEN J	2015	1	8	0.8
ELLIESEN J	2016	1	1	0.111
FLAMMINI A	2015	1	11	1.1
FLAMMINI A	2016	1	11	1.222
NOVELLA JL	2021	1	0	0
NOVELLA JL	2022	1	0	0
SANCHEZ S	2021	1	0	0
SANCHEZ S	2022	1	0	0
VEZZOLI A	2015	1	11	1.1
VEZZOLI A	2016	1	11	1.222

On the other hand, ELLIESEN J experienced a noticeable decline in citation impact, with a drop from 8 citations in 2015 to just 1 in 2016. Additionally, CORTES U's older publications, from 2013 and 2019, have had limited impact, with very few or no citations, highlighting the challenges of maintaining academic relevance over time. Overall, the data underscores the varying degrees of influence these authors' works have had within the academic community.

### 4.3 Corresponding author's countries and countries citation

**Fig. 4:**
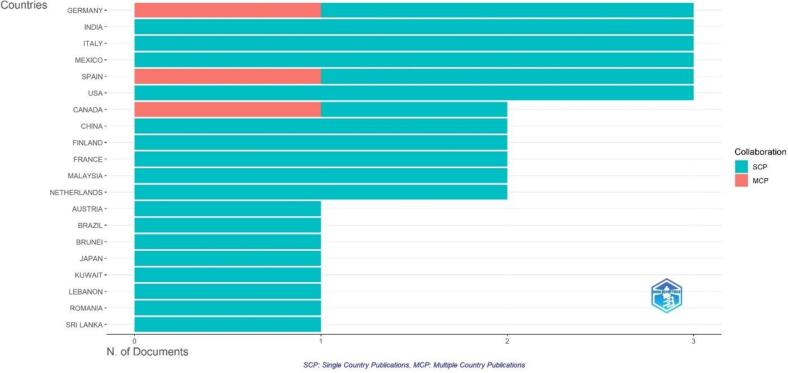
Corresponding author's countries.

The results of the analysis are presented in Fig. 4, which displays the number of articles, the Social Content Performance (SCP) score, the Media Content Performance (MCP) score, the frequency, and the MCP ratio for each country. The table 2 is sorted in descending order based on the number of articles.

**Table 2:** Country citation.

Country	TC	Average Article Citations
Canada	43	21,50
Australia	28	28,00
Netherlands	23	11,50
Italy	22	7,30
Mexico	18	6,00
Brunei	13	13,00
Finland	12	6,00
Austria	11	11,00
Germany	9	3,00
Spain	9	3,00
Lebanon	7	7,00
Malaysia	5	2,50
United Kingdom	4	4,00
China	3	1,50
India	3	1,00
USA	2	0,70
Japan	1	1,00
Romania	1	1,00
Brazil	0	0,00
France	0	0,00
Kuwait	0	0,00
Sri Lanka	0	0,00

The analysis revealed that Germany, India, Italy, Mexico, Spain, and the USA were the most relevant countries in terms of the number of articles published. Each of these countries had three articles associated with them, indicating their significance in the context of the study.

When considering the SCP score, India, Italy, Mexico, and the USA received a perfect score of 3, indicating high social content performance. Germany and Spain received a slightly lower score of 2, suggesting a relatively lower social content performance compared to the other countries.

In terms of the MCP score, Canada, Germany, and Spain were the only countries to receive a score of 1, indicating a higher media content performance. The remaining countries had an MCP score of 0, suggesting a lack of media content performance.

The frequency of articles for each country was calculated by dividing the number of articles by the total number of articles in the dataset. All countries had a frequency of 0.075, indicating an equal representation in the dataset.

The MCP ratio, which represents the ratio of media content performance to the total number of articles, was calculated for each country. Canada had the highest MCP ratio of 0.5, followed by Germany and Spain with an MCP ratio of 0.333. The remaining countries had an MCP ratio of 0, indicating a lack of media content performance relative to the number of articles.

The results suggest that Germany, India, Italy, Mexico, Spain, and the USA were the most relevant countries in terms of the number of articles published. However, when considering social content performance, India, Italy, Mexico, and the USA outperformed Germany and Spain. In terms of media content performance, Canada, Germany, and Spain had a higher performance compared to the other countries. These findings provide valuable insights into the representation and performance of different countries in the context of the study.

### 4.4 Production distribution by continent

**Fig. 5:**
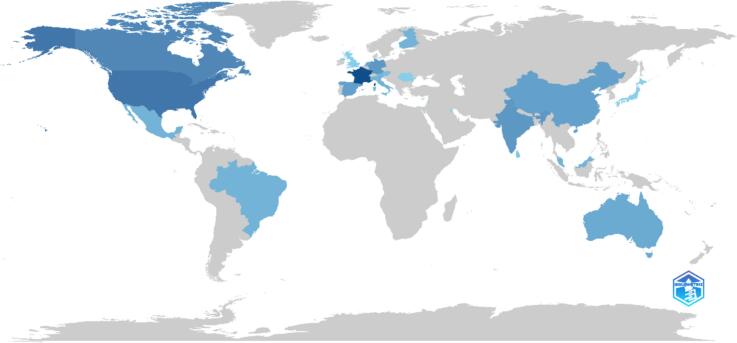
Country scientific production.

In this section, we present the results of our analysis of various countries' production and categorize them into continents. The data used for this analysis includes each country's production frequency, as shown in Fig. 5.

#### 4.4.1 Europe

In Europe, France leads with the highest production frequency at 16 instances, followed by Germany with 7 instances and the Netherlands with 5. Other countries such as Finland, Italy, Austria, Romania, and the UK each have a production frequency of 4 or less. This indicates that while France is the dominant producer, several other European countries also contribute to the overall production landscape.

#### 4.4.2 North America

In North America, the USA is the leading country with 11 production instances, followed closely by Canada with 9 instances. This demonstrates a strong production presence in the continent, particularly in the USA, which significantly outpaces other North American countries in production frequency.

#### 4.4.3 Asia

Asia presents a diverse production landscape with India and China each having a production frequency of 7, making them the leading countries in the continent. Spain follows closely with 6 instances, while Australia and Malaysia have 5 and 4 instances, respectively. Other countries such as Brunei, Sri Lanka, Japan, Kuwait, and Lebanon have a production frequency of 2 or less, showing a varied but active production environment in Asia.

#### 4.4.4 South America

In South America, Brazil and Mexico both have a production frequency of 4, making them the top producers on the continent. Compared to Europe, North America, and Asia, South America has a relatively lower production frequency, highlighting a less intensive production activity in this region.

Europe and North America have the highest production frequencies, with France and the USA being the leading countries. Asia also demonstrates significant production levels, with India and China as the prominent contributors. South America, on the other hand, has a relatively lower production frequency compared to the other continents. It is important to note that these results are based on the frequency of production instances and do not reflect the actual quantity or value of production. Further analysis would be required to determine the significance of these production frequencies in relation to the overall economic contributions of each country and continent.

### 4.5 Thematic map clusters

**Fig. 6:**
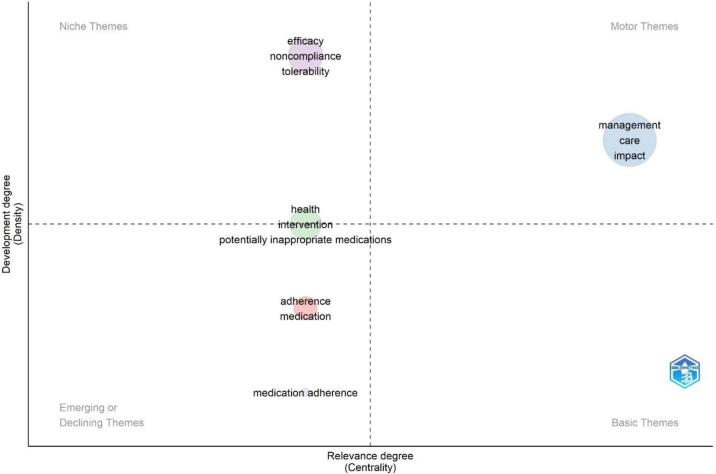
Thematic map cluster.

Fig. 6 presents the results of the bibliometric analysis on thematic map clusters. The analysis focused on five clusters: adherence, management, health, efficacy, and medication adherence. The metrics used to measure the characteristics of these clusters were Callon Centrality, Callon Density, Rank Centrality, Rank Density, and Cluster Frequency.

#### 4.5.1 Cluster: Adherence

The cluster “adherence” had a Callon Centrality score of 0, indicating it did not have a central position in the network. However, it had a relatively high Callon Density score of 62.5, suggesting solid connections among the publications within this cluster. Regarding Rank Centrality, the cluster scored 2.5, indicating a moderate level of centrality. The Rank Density score was 2, suggesting some dense areas within the cluster. The Cluster Frequency score for “adherence” was 4, indicating that this cluster appeared frequently in the literature.

#### 4.5.2 Cluster: Management

The “management “cluster had a Callon Centrality score of 1.1, indicating a slightly higher centrality than the “adherence” cluster. The Callon Density score for this cluster was 77.86, suggesting a high level of connectivity among the publications. Regarding Rank Centrality, the cluster scored 5, indicating a relatively high centrality. The Rank Density score was 4, suggesting the presence of dense areas within the cluster. The Cluster Frequency score for “management” was 17, indicating a high frequency of appearance in the literature.

#### 4.5.3 Cluster: Health

The cluster “health” had a Callon Centrality score of 0, indicating a lack of central position in the network. However, it had a relatively high Callon Density score of 75, suggesting strong connections among the publications within this cluster. Regarding Rank Centrality, the cluster scored 2.5, indicating a moderate level of centrality. The Rank Density score was 3, suggesting the presence of some dense areas within the cluster. The Cluster Frequency score for “health” was 6, indicating a moderate frequency of appearance in the literature.

#### 4.5.4 Cluster: Efficacy

The cluster “efficacy” had a Callon Centrality score of 0, indicating a lack of central position in the network. However, it had a relatively high Callon Density score of 83.33, suggesting strong connections among the publications within this cluster. Regarding Rank Centrality, the cluster scored 2.5, indicating a moderate level of centrality. The Rank Density score was 5, suggesting the presence of some dense areas within the cluster. The Cluster Frequency score for “efficacy” was 7, indicating a moderate frequency of appearance in the literature.

#### 4.5.5 Cluster: Medication adherence

The cluster “medication adherence” had a Callon Centrality score of 0, indicating a lack of central position in the network. It had a Callon Density score of 50, suggesting moderate connectivity among the publications within this cluster. Regarding Rank Centrality, the cluster scored 2.5, indicating a moderate level of centrality. The Rank Density score was 1, suggesting the presence of some dense areas within the cluster. The Cluster Frequency score for “medication adherence” was 2, indicating a relatively low frequency of appearance in the literature.

The results of the bibliometric analysis revealed variations in the characteristics of the thematic map clusters. The “management” cluster had the highest centrality scores across all metrics, indicating its importance and influence in the network. The “adherence,” “health,” and “efficacy” clusters had similar centrality scores, suggesting their comparable significance. The “medication adherence” cluster had lower centrality scores, indicating its relatively lesser importance in the network. These findings provide insights into the structure and dynamics of the research landscape surrounding these thematic map clusters.

### 4.6 Cluster analysis results from the dataset

Table 3 present the cluster analysis results from the dataset. The analysis focused on identifying clusters of words based on their occurrences in the text. Each word was assigned to a cluster, and the clusters were labelled based on the dominant theme represented by the words within them. Additionally, three centrality measures, namely betweenness centrality, closeness centrality, and PageRank centrality, were calculated for each word within its respective cluster.

**Table 3:** Thematic cluster.

Occurrences	Words	Cluster	Cluster_Label	btw_centrality	clos_centrality	pagerank_centrality
2	adherence	1	adherence	135	0,033333333	0,016216635
2	medication	1	adherence	108	0,03030303	0,015208548
5	management	2	management	864,3,333,333	0,011494253	0,031588021
2	care	2	management	0	0,007518797	0,008283408
2	impact	2	management	318	0,009009009	0,015387276
2	mortality	2	management	142,3,333,333	0,008928571	0,015792654
2	nonadherence	2	management	318	0,009009009	0,015387276
2	people	2	management	292,3,333,333	0,01	0,015984619
2	system	2	management	0	0,007751938	0,008743983
2	health	3	health	0	0,006410256	0,008569653
2	intervention	3	health	235,3,333,333	0,008695652	0,014387237
2	potentially inappropriate medications	3	health	203,6,666,667	0,008403361	0,015618323
3	efficacy	4	efficacy	116	0,043478261	0,020616547
2	noncompliance	4	efficacy	8	0,024390244	0,013190294
2	tolerability	4	efficacy	8	0,024390244	0,013190294
2	medication adherence	5	medication adherence	0	0,006097561	0,007622733

#### 4.6.1 Cluster 1: Adherence

The first cluster, labelled as “adherence,” consists of two words: “adherence” and “medication.” Both words appeared twice in the text. The betweenness centrality measure for “adherence” is 135, indicating its importance in connecting other words in the text. The closeness centrality measure for “adherence” is 0.033, suggesting its proximity to other words in the text. The PageRank centrality measure for “adherence” is 0.016, indicating its importance in the overall network of words.

#### 4.6.2 Cluster 2: Management

The second cluster, “management,” includes several words related to healthcare management. These words are “management,” “care,” “impact,” “mortality,” “nonadherence,” “people,” “system,” and “medication.” The word “management” appeared five times, while the other words appeared twice each. The betweenness centrality measure for “management” is 864.33, indicating its significant role in connecting other words. The closeness centrality measure for “management” is 0.011, suggesting its proximity to other words. The PageRank centrality measure for “management” is 0.032, highlighting its importance in the overall network.

#### 4.6.3 Cluster 3: Health

The third cluster, labelled as “health,” consists of the words “health,” “intervention,” and “potentially inappropriate medications.” Each word appeared twice in the text. The betweenness centrality measure for “health” is 0, indicating its lack of influence in connecting other words. The closeness centrality measure for “health” is 0.007, suggesting its relative proximity to other words. The PageRank centrality measure for “health” is 0.009, indicating moderate importance in the overall network.

#### 4.6.4 Cluster 4: Efficacy

The fourth cluster, labelled as “efficacy,” includes the words “efficacy,” “noncompliance,” and “tolerability.” The word “efficacy” appeared three times, while the other words appeared twice each. The betweenness centrality measure for “efficacy” is 116, indicating its significance in connecting other words. The closeness centrality measure for “efficacy” is 0.043, suggesting its relative proximity to other words. The PageRank centrality measure for “efficacy” is 0.021, highlighting its importance in the overall network.

#### 4.6.5 Cluster 5: Medication adherence

The fifth cluster, “medication adherence,” consists of the phrase “medication adherence.” This phrase appeared twice in the text. The betweenness centrality measure for “medication adherence” is 0, indicating its lack of influence in connecting other words. The closeness centrality measure for “medication adherence” is 0.006, suggesting its relative proximity to other words. The PageRank centrality measure for “medication adherence” is 0.008, indicating moderate importance in the overall network.

The cluster analysis revealed distinct clusters of words based on their occurrences in the text. The clusters were labelled based on the dominant theme represented by the words within them. The centrality measures provided insights into the importance and influence of each word within its respective cluster. These findings contribute to a better understanding of the relationships between words in the text and can inform future research.

### 4.7 Annual production of robot drug dispenser and citable years

The annual production and citable years of articles published from 1999 to 2023 were analyzed and discussed. The data presented in Table 4 show the number of articles published each year (N), the mean total citations per article (MeanTCperArt), the mean total citations per year (MeanTCperYear), and the number of citable years (CitableYears).

**Table 4:** Annual production of Robot Drug Dispenser and citable years.

Year	N	MeanTCperArt	MeanTCperYear	CitableYears
1999	1,00	28	1,08	26
2006	2,00	5,5	0,29	19
2007	1,00	4	0,22	18
2010	2,00	7	0,47	15
2012	2,00	13,5	1,04	13
2013	3,00	12,67	1,06	12
2014	1,00	4	0,36	11
2015	2,00	9,5	0,95	10
2016	4,00	3,25	0,36	9
2017	2,00	1,5	0,19	8
2018	4,00	1,75	0,25	7
2019	3,00	1,67	0,28	6
2020	5,00	7,8	1,56	5
2021	3,00	0,67	0,17	4
2022	2,00	0	0,00	3
2023	3,00	0	0,00	2

The annual production and citable years analysis reveals several interesting patterns and trends.

First, the number of articles published each year varied across the analyzed period. The highest number of articles was published in 2020, with five. On the other hand, 1999, 2007, 2014, and 2022 had the lowest number of publications, with only one article each.

Next, the mean total citations per article (MeanTCperArt) and the mean total citations per year (MeanTCperYear) were calculated to assess the impact and productivity of the published articles. The mean total citations per article ranged from a minimum of 0 to a maximum of 28. In 1999, we had the highest average citations per article, with an impressive mean of 28 citations. In contrast, 2021 and 2022 had the lowest average citations per article, with mean values of 0.67 and 0, respectively.

Similarly, the mean total citations per year ranged from a minimum of 0 to a maximum of 1.56. 2020 had the highest average citations per year, with a mean value of 1.56. Conversely, 2021 and 2022 had the lowest average citations per year, with mean values of 0.17 and 0, respectively.

Furthermore, the number of citable years (CitableYears) indicates the years the articles published in a specific year remain citable. The range of citable years varied from a minimum of 2 years to a maximum of 26 years. 1999 had the highest number of citable years, with articles remaining citable for an average of 26 years. In contrast, 2022 and 2023 had the lowest number of citable years, with articles remaining citable for only 3 and 2 years, respectively.

The analysis of the annual production and citable years demonstrates variations in the number of articles published, their impact in terms of citations, and the longevity of citable articles across the analyzed period. These findings provide valuable insights into the productivity and impact of articles published in the field over time.

## 5 Discussions

The findings of this study offer a detailed examination of the research landscape surrounding robotic tablet dispensing machines for elderly care, with a particular emphasis on the trends in research growth, collaboration patterns, thematic focus, and geographic disparities.

### 5.1 Research growth and impact

The increasing research output in this field, with an annual growth rate of 4.68 %, reflects a growing awareness of the importance of robotic technologies in addressing the healthcare needs of the elderly. This growth aligns with global trends of aging populations and the consequent demand for improved healthcare solutions. However, the relatively modest citation rate of 5.35 per document suggests that while the topic garners interest, it may not yet have achieved significant influence within the broader scientific community. This could be attributed to the field's relatively young age, as indicated by the average document age of 7.8 years. Additionally, the research may still be in a preliminary stage, focusing on technological development rather than on large-scale implementation and evaluation, which are typically associated with higher citation rates.

### 5.2 Collaboration patterns

The field's high level of collaboration, with an average of 5.12 co-authors per document, highlights its multidisciplinary nature, drawing on expertise from fields such as robotics, geriatrics, pharmacy, and health informatics. This interdisciplinary collaboration is crucial for advancing complex healthcare technologies.^35^ However, the low rate of international collaboration (7.5 % of documents) indicates a significant limitation. The lack of global collaboration may hinder the exchange of diverse perspectives and slow the adoption of innovative solutions across different healthcare systems. Increasing international partnerships could enhance the development and dissemination of these technologies, potentially leading to more globally relevant innovations.

### 5.3 Thematic focus and trends

The thematic analysis identified five primary research clusters: adherence, management, health, efficacy, and medication adherence. The “management” cluster, with the highest centrality scores, emerges as the most influential, underscoring the critical role of effective management strategies in the deployment of robotic dispensing machines.^36^ This finding suggests that while the technology itself is important, its successful implementation heavily depends on how it is managed within healthcare settings. The strong focus on “adherence” and “efficacy” reflects ongoing efforts to address the common challenges of medication adherence among the elderly, which has been identified as a key factor in improving health outcomes.^37^

Interestingly, the “medication adherence” cluster, despite its relevance, has lower centrality scores, indicating that it may not be as central to the research discourse as other themes. This could point to a gap in the literature where more targeted studies on the direct impact of robotic dispensing machines on medication adherence are needed.^38^ Addressing this gap could provide more concrete evidence of the benefits of these technologies, potentially leading to wider adoption in clinical practice.

### 5.4 Geographic and continental disparities

The analysis of research distribution by continent reveals a concentration of studies in Europe and North America, with France and the USA leading in their respective regions. This concentration could reflect the higher levels of investment in healthcare technology research and the advanced infrastructure available in these regions. In contrast, the lower research output from South America suggests an underrepresentation that could be due to limited resources or differing healthcare priorities. Asia, particularly India and China, shows a growing interest, likely driven by their large aging populations and the increasing burden of chronic diseases.

The geographic disparities in research output highlight the need for more inclusive research efforts that encompass diverse healthcare settings and populations. Expanding research efforts to underrepresented regions could lead to the development of more adaptable and culturally relevant robotic dispensing technologies.

### 5.5 Implications and future directions

The findings of this study suggest several important implications for future research and development in the field of robotic tablet dispensing machines. First, there is a clear need for increased international collaboration to accelerate innovation and ensure that these technologies are globally applicable. Second, while the current research focus on management and adherence is essential, future studies should also prioritize practical evaluations of the efficacy of these technologies in real-world settings. Such studies could provide the evidence needed to drive wider adoption and integration into healthcare systems. Finally, addressing the geographic disparities in research output by encouraging more studies in underrepresented regions could enhance the field's relevance and impact. By fostering a more globally inclusive research environment, the field can develop technologies that are not only innovative but also accessible and effective across diverse healthcare systems.

## 6 Conclusion

The bibliometric analysis highlights the growing and evolving research landscape of robotic tablet dispensing machines for elderly care. The findings indicate a robust interest in improving medication management and adherence, with a steady increase in research output and a moderate citation impact. Collaborative, multidisciplinary approaches are prevalent, though international collaboration remains limited. The analysis underscores the importance of continuing to develop and disseminate research in this field, particularly through peer-reviewed journals to enhance impact. Increasing global cooperation could further enrich the research landscape, fostering innovation and sharing best practices across borders. The thematic clusters identified, particularly the prominence of management-related research, reflect the practical challenges and priorities in integrating robotic dispensing systems into healthcare. Future research should continue to address these practical aspects while also exploring under-researched areas to provide comprehensive solutions for elderly care. Overall, this study provides a valuable foundation for understanding the current state of research on robotic tablet dispensing machines and identifies key areas for future investigation and collaboration.

## Credit authorship contribution statement

OSA put forward the idea and designed the study. All collected the literature and wrote the manuscript. OSE, ABW and WMN revised the manuscript. OSA prepared the figures and tables. All authors contributed to data analysis, drafting, and critically revised the study, agreed to be accountable for all aspects of the work, read, and approved the final manuscript.

## Funding

Authors funded this research.

## Declaration of competing interest

The authors report no conflicts of interest in this work.
